# Spatial, Temporal, and Dynamic Behavior of Different Entropies in Seismic Activity: The February 2023 Earthquakes in Türkiye and Syria

**DOI:** 10.3390/e27050462

**Published:** 2025-04-25

**Authors:** Denisse Pastén, Eugenio E. Vogel, Gonzalo Saravia, Antonio Posadas

**Affiliations:** 1 Departamento de Física, Facultad de Ciencias, Universidad de Chile, Santiago 9170022, Chile; 2 Departamento de Física, Universidad de La Frontera, Casilla 54-D, Temuco 4811230, Chile; 3 Facultad de Ingeniería, Universidad Central de Chile, Santiago 8330601, Chile; 4 Los Eucaliptus 1189, Temuco 4812537, Chile; gonzalo.saravia@gmail.com; 5 Departamento de Química y Física, Universidad de Almeria, 04120 Almeria, Spain; aposadas@ual.es; 6 Instituto Andaluz de Geofísica, Campus Universitario de Cartuja, Universidad de Granada, 18071 Granada, Spain

**Keywords:** entropy analysis, Türkiye eartrhquake, spatio-temporal analysis of seismicity

## Abstract

Türkiye and Syria were hit by two powerful earthquakes on 6 February 2023. A 7.5 magnitude earthquake, soon followed by a second 7.4 magnitude seism, devastated the area. The present study compares three different entropies using data from 2017 to 2023 (55,823 events) in this region and is the first study to use Shannon entropy, Tsallis entropy, and mutability for analyzing the seismic activity in this region. A couple of years before these large earthquakes, both Shannon entropy and mutability show an overall decrease, potentially indicating upcoming large events; however, the detailed results on mutability offer an advantage, as discussed in this paper. A simultaneous overall increase in Tsallis entropy may also point to some kind of warning of the possible occurrence of large events in the area a couple of years later. The three entropies show how they are presently slowly recovering to previous levels in the affected areas. Longer-term studies combining complementary entropies could help to determine regional seismic risk.

## 1. Introduction

One of the great challenges of modern seismology is characterizing the chaotic and non-linear behavior of earthquakes of any size and in any environment, whether tectonic or volcanic. No direct observations of stresses at different depths underground are possible, so the data refer to measurements on the surface of the planet. Each earthquake originates a chain of aftershocks of smaller magnitude, which are thus correlated. Therefore, every significant earthquake triggers a series of related aftershocks. However, the largest earthquakes may not be related, so regional seismic data typically include both the connected and unconnected events, contributing to earthquake unpredictability. Advancements in identifying special behaviors or premonitory signs before a major earthquake require different methods and techniques. Here, we attempt an entropic description of the different regimes present in the region where a pair of large earthquakes devastated regions in Türkiye and Syria on 6 February 2023.

The complete description (physical, mathematical, and geological) of the seismic phenomenon, based on direct observations, would make it possible to design a model of earthquake occurrence that could indicate where an important earthquake could occur. The exact locations and dates of future earthquakes are more elusive. Given that our current knowledge does not allow us to develop such models, the seismic forecasting analysis has relied on the study of certain precursors, phenomena associated with the occurrence of an event that could indicate the imminence of an earthquake. Thus, the emissions of radon gas (e.g., Omori et al., 2021 [1]) or $CO_{2}$ (e.g., D’Incecco et al., 2021 [2]) before an earthquake, the electromagnetic radiation of certain frequencies detected before an earthquake (e.g., Rabinovitch et al., 2017 [3]) or the changes in the $V_{p}/V_{s}$ ratio (e.g., Placinta et al., 2022 [4]) are precursor phenomena associated in the scientific literature with the causality of an earthquake.

However, earthquakes are precise physical phenomena associated with the exchange of energy during accumulation and deformation processes, as well as during rupture and propagation processes as seismic waves. Earthquakes make up a physical system that transitions from near-equilibrium to non-equilibrium. This system continuously undergoes variations in internal energy and presents difficulties when analyzed using stochastic processes. On this basis, the laws of thermodynamics and, fundamentally, the Second Law, or the use of entropy, are useful tools to describe this phenomenon [5,6].

We propose using entropy to show the equilibrium state of a seismically active region (seismic system). Investigating the relationship between entropy variations and large earthquakes may provide insights into earthquake prediction by recognizing the onset of irreversible transitions. From this point of view, the seismic system evolves from an unstable initial state (owing to external stresses) to another, where the stresses drop (at least partially) after the earthquake and its aftershock regime occurs. It is an irreversible transition that entails a sudden variation in entropy.

In this work, we present a study comparing the following three entropy forms to appreciate the behavior of the mechanisms that unleash large earthquakes: Shannon entropy, Tsallis entropy, and mutability (or a form of dynamic entropy). The application is over the dataset associated with the seismic activity in Türkiye, where the entropy variations are mainly studied based on the earthquake magnitudes, with just an initial application on time inter-events. The area chosen was affected by the Mw7.5 and Mw7.4 earthquakes in Türkiye and Syria on 6 February 2023, followed by an intense aftershock regime. To the best of our knowledge, this is the first study to address entropy in this area and to compare three entropies in a zone other than a subduction, in this case, the entropy study of the earthquakes that occurred in Türkiye and Syria in the years 2020 and 2023 show a behavior similar to that previously found in subduction zones.

Since the different entropy functions are sensitive to changes in seismic activity, they can be used for its characterization. Moreover, they behave differently (they increase or decrease under critical conditions) and are complementary to each other, allowing for a more comprehensive description of the phenomena; in particular, we will show how they combine to illustrate the contrast between the foreshock and aftershock regimes.

## 2. Data

Türkiye and Syria are located on the Anatolian and Arabian plates, respectively. The East Anatolian Fault Zone bounds the Anatolian Plate in the East. A left-lateral strike-slip fault represents a plate boundary extending over 500 km between the Anatolian and Arabian plates (where Syria is located) (Bulut et al., 2012 [7]), where surprising rupture trajectories have been detected (Zhe Jia et al. (2023) [8]). Continually, the slip rate across the East Anatolian Fault Zone varies between 6 and 10 mm/year, following different measurement techniques (e.g., Taymaz et al., 1991 [9]; McClusky et al., 2000 [10]; Orgülü et al., 2003 [11]). At times, the strain accumulates and is intermittently released by occasional earthquakes at a magnitude of seven or more (Dal Zilio and Ampuero, 2023 [12]).

On 6 February 2023, Türkiye and Syria were hit by two devastating earthquakes (Jiang et al., 2023 [13]). First, a $M_{w}=$ 7.5 earthquake, generated by the rupture of the southeastern part of the East Anatolian Fault Zone (Lekkas et al., 2023 [14]), was located at 37.16 North Latitude, 37.09 East Longitude, westward from Gaziantep city. The first event probably triggered the generation of a new $M_{w}=$ 7.4 earthquake only 9 h later within the same fault zone; it was situated at 38.08 North Latitude, 37.18 East Longitude, approximately 100 km further north.

Over 600 aftershocks were recorded within 24 h of the $M_{w}$ 7.5 earthquake. Remarkably, only 11 min after the main shock, an aftershock of $M_{w}$ 6.7 occurred. Moreover, 25 aftershocks measuring *M* 4.0 or greater were recorded within 6 h of the main tremor. On the other hand, the $M_{w}$ 7.4 earthquake triggered its aftershock sequence, with two aftershocks exceeding magnitude 6.0 (http://www.koeri.boun.edu.tr/sismo/2/en/, accesed on 24 December 2024).

**Figure 1:**
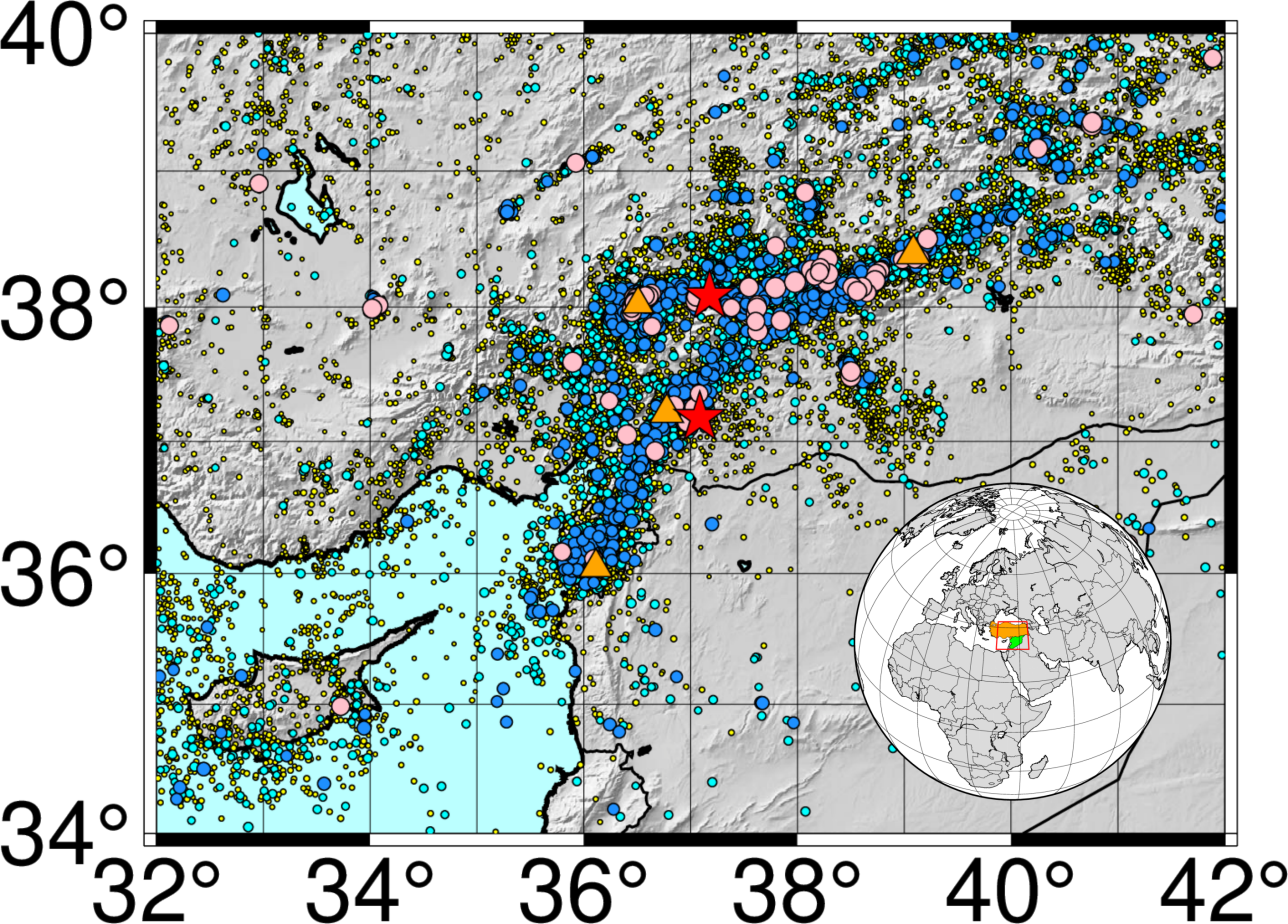
Map showing the epicenters of the study area. The rectangle of the analyzed area has been marked on the globe. The countries of Türkiye (orange) and Syria (green) have also been marked on the map. The yellow circles correspond to earthquakes with a magnitude between 2.0 and 2.9, the cyan circles are those with a magnitude between 3.0 and 3.9, the blue circles are those with a magnitude between 4.0 and 4.9, the pink circles are those with a magnitude between 5.0 and 5.9, the orange triangles are those with a magnitude between 6.0 and 6.9 and, finally, the red stars correspond to the first large earthquake Mw7.5 (further south) and the second large earthquake Mw7.4 (further north), which occurred just 9 h after the first.

For the present study, we extract data from the catalogs of the Kandilli Observatory and Earthquake Research Institute (KOERI) and the Regional Earthquake and Tsunami Monitoring Center (RETMC) [15]. The RETMC is currently capable of real-time seismic data analysis and the rapid delivery of earthquake parameters and tsunami early warning information to all disaster-related organizations in an efficient and reliable manner. Earthquakes occurring in any part of Türkiye are located, and their magnitude is calculated in near-real time using the data received from this network. Currently, the network comprises a total of 238 stations connected to the RETMC via satellite, GPRS, or internet connection. The data for this paper were selected within the “rectangle” 34 N to 40 N and 32 E to 42 E, from 1 January 2017 to 31 December 2023, initially providing 74,542 earthquakes to be considered. Table 1 lists the characteristics of the main recent earthquakes within the geographical area defined in Figure 1. The largest triggering earthquakes in each sequence were chosen to illustrate this activity.

**Figure 2:**
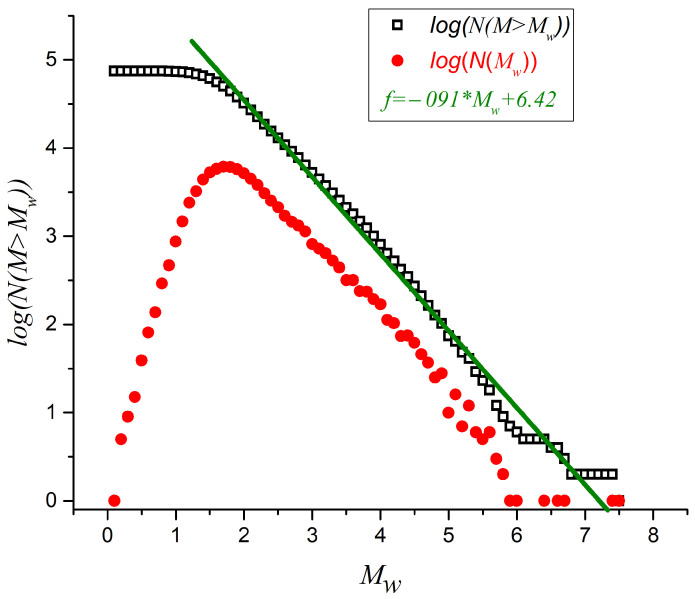
Gutenberg–Richter relationships for the original set of 74,542 seismic events recorded within the rectangle defined in Figure 1.

We begin the analysis by presenting the basic properties of the magnitude sequence. Figure 2 considers the Gutenberg–Richter analysis based on the 74,542 earthquakes with an epicenter within the geodesical coordinates defined in Figure 1. The linear fit of the decay of the magnitude abundance corresponds to a straight line described by y=A−bx, where *x* is the magnitude *M* of the seism, *y* is the number of earthquakes with a magnitude larger than *M*, A is the intercept on the ordinate axis, and *b* is the slope (a positive number in this formulation), which happens to be 0.91 in this case. The completitude magnitude (determined by the point of maximum curvature) is $M_{c}=1.6$. After filtering for earthquakes that are equal or over this $M_{C}$ value, we are left with 55,853 earthquakes for the remaining analysis.

**Figure 3:**
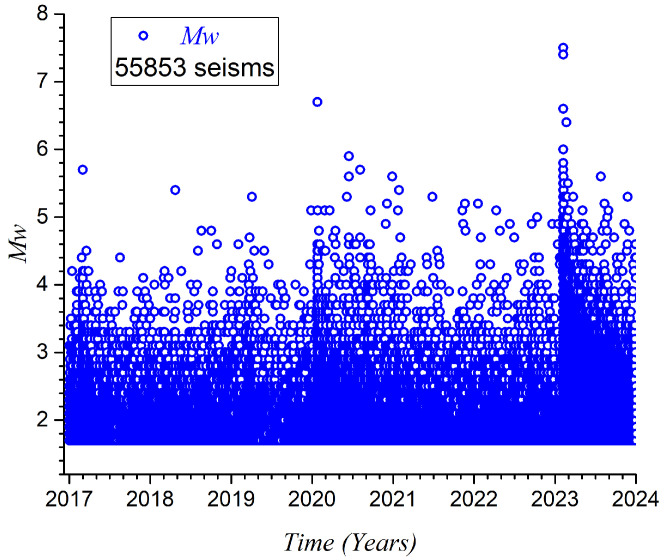
Magnitude vs. time for set of 55,853 earthquakes after GR analysis. Time spans seven years, beginning on 1 January 2017.

**Figure 4:**
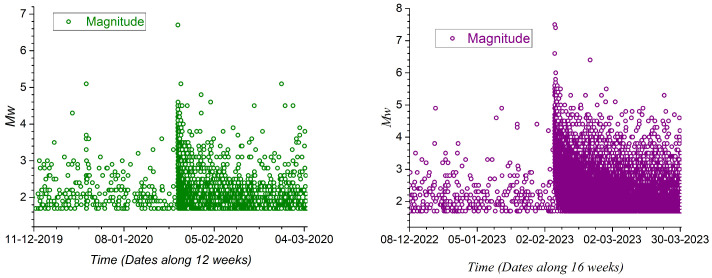
(**Left**) Magnitude vs. time of foreshocks and aftershocks related to *M* 6.7 earthquake on 24 January 2020. (**Right**) Magnitude vs. time of foreshocks and aftershocks related to Mw 7.5 earthquake on 16 July 2023. Time grouped in weeks around main earthquakes.

The sequence of these magnitudes is illustrated in Figure 3, while the details associated with the two important triggering earthquakes highlighted in Table 1 are shown in Figure 4. We can observe two transitions in the number of events close to days 1100 and 2190. These transitions coincide with the occurrence of earthquakes of magnitudes 6.7 and 7.5, respectively. Following the 7.4 earthquake, the aftershock regime is superposed on the one with a magnitude of 7.5 occurring nearby on the same day.

## 3. Theoretical Basis

In this section, we briefly summarize the concepts and ideas behind each of the entropic functions used in the present article. Interested readers are kindly invited to review the theoretical derivations and examples given in the cited references. In particular, Shannon entropy and mutability were presented, compared, and applied to the seismic activity in Iquique (Chile). In contrast, Tsallis entropy and mutability were described in detail, compared, and used to discuss the seismic activity in Alaska [16].

### 3.1. Shannon Entropy

In 1948, Shannon proposed a form of entropy derived from information theory [17]. If $p_{i}$ is the probability that a system is in state *i*, then the Shannon entropy is given by the following:

(1) $$H=-\sum_{i}p_{i}\;ln\left(p_{i}\right)\;.$$

It can be formally extended to other statistical physics analyses. The exact determination of the probabilities $p_{i}$ is limited to small systems only [18]. Therefore, for most systems, it is necessary to sample a large number of instances to obtain the probability $p_{i}$ by the ratio of the number $n_{i}$ of instances when the state *i* is visited over the total number of visits *N*, which is expressed as follows:

(2) $$p_{i}=\frac{n_{i}}{N},$$

with

(3) $$\sum_{i}p_{i}=1.0\;.$$

One way to achieve this parameter is to organize the data in a vector file. From there, a histogram can be obtained, rendering the number of times each magnitude appears (frequency or abundance), which leads directly to the probabilities $p_{i}$ by Equation (2), allowing us to obtain the Shannon entropy by Equation (1). Other ways of calculating these probabilities are possible, but in general, they reflect the normalized probability of obtaining a certain value for the observable [17,19]. In the present article, we will use the previously described method.

### 3.2. Mutability

The same vector file *V* used to calculate *H* in the previous subsection, weighting *w* bytes, is now treated with a data compressor *wlzip* [20,21]. This data compressor is tailored to recognize digit chains of a given length at precise positions within numerical registers, giving rise to its full name, *word length zipper* (wlzip for short). This is very useful for recognizing meaningful numerical data associated with properties of the system in a time-series analysis.

With wlzip, a new file $V^{*}$ is created containing the same information of *V*, but in a coded way. This new file, weighting $w^{*}$ bytes, is a map of the previous one, keeping track of the values and their location in the original file. Each new value in *V* opens a new row in $V^{*}$. Successive appearances of such values are denoted by the distance to the last appearance; repetitions are denoted by the number of them after a comma. In summary, each different value of the observable appears only once in the map, written to the left in a row of $V^{*}$, followed by the relative positions in *V*, written to the right in this row. Then, it is easy to find the frequency $n_{i}$ of the appearances of value $v_{i}$ by the number of its appearances stored in $V^{*}$. This leads to the normalized probability $p_{i}$ of obtaining the value $v_{i}$, as given by Equation (2), where *N* is the total number of registers in *V*. In this way, Shannon entropy can be considered as a particular static case of mutability, when the dynamical aspects of the distribution of values in *V* are ignored. However, mutability bears more information than Shannon entropy, as such dynamical aspects are embedded in the positions and repetitions stored in each row of $V^{*}$. Thus, if *wlzip* detects frequent repetitions of several values in *V*, then ζ remains low; if the same values are mixed with sparse repetitions, then ζ is larger.

As a result of the mutability technique, we obtain a new file, which is a map of the original file, keeping track of the relative positions of the registers grouped by identical values within predefined numerical precision. This map allows us to reconstruct the original file, ensuring that no information is lost (this procedure will not be necessary here). The weight of the map file is $w^{*}$. A detailed example of this procedure for magnitudes of real earthquakes can be found in Table 1 of [21].

Then, the mutability ζ(t) is defined by the following:

(4) $$\zeta =\frac{w^{*}}{w}\;.$$

Let us summarize the meaning of mutability: When only a few states are accessible, the corresponding property values are frequently repeated, and the map is short, resulting in low mutability values. Conversely, when multiple states are accessible, repetitions are rare, resulting in a chaotic regime with a large $w^{*}$ and high values of mutability.

### 3.3. Tsallis Entropy

In simple terms, the Tsallis entropy can be calculated using the following expression [22,23,24]:

(5) $$S_{q}=\frac{1}{q-1}\left(1-\sum_{i=1}^{\Omega }p_{i}^{q}\;.\right)$$

where the probabilities of getting the *i*-th value for the observable can be the same as those already discussed when presenting Shannon entropy. The sum is over all the states *i* belonging to the ensemble Ω, which describes all the possible magnitudes of the seismic events. The value of *q* can be obtained from the Gutenberg–Richter slope *b* by means of the following [16,22,25]:

(6) $$b=2\frac{2-q}{q-1}.$$

From here, we can see that, in general, 1.0≤q≤2.0. However, in seismology, slope b leads to q>1.0, indicating that the data are correlated, and the entropy is non-additive and far from Boltzmann–Gibbs entropy that is recovered in the limit q→1 (see, for instance, the Appendix A in Flores-Márquez et al. (2024) [26]).

## 4. Methodology

To gain dynamical insights into the seismic activity, we will consider the overlapping windows of *W* consecutive events over the 7 years analyzed in the present study. It should be noted that this does not imply the same time span for all windows, but it assures the same number of registers to be considered in the statistical analysis. Thus, the first window is from event number 1 to event number *W*, the second window is from event number 2 to event number W+1, and so on. The time associated with each window will be the time of the last event of the corresponding time window.

Two series of data arise naturally to be considered for the statistical analysis—magnitude sequence and inter-event sequence. We will focus on magnitudes in the present article for homogeneity and space reasons, but we will still include one figure with results on the inter-event sequence to show its feasibility.

A vector file with the sequence of all the magnitudes extracted from the catalog, expressed in the format I.D, where *I* is an integer digit and *D* is a decimal digit, is prepared. This sequence is then dynamically analyzed with windows comprising *W* consecutive events to obtain ζ(t), H(t), and $S_{q}\left(t\right)$ based on magnitudes.

## 5. Results and Discussion

We will present the results in the following order: Mutability on magnitudes; Mutability and Shannon entropy on magnitudes; Tsallis entropy on magnitudes; and Mutability and Tsallis entropy on magnitudes.

We begin the analysis by considering the Gutenberg–Richter plot of the 74,542 earthquakes with an epicenter within the geodesical coordinates defined in Figure 1. This is presented in Figure 2, with a completitude magnitude of $M_{c}=1.6$. After purging all the earthquakes with a magnitude under $M_{c}$, we are left with 55,853 earthquakes for the remaining analysis. In addition, Figure 2 also shows the negative slope of the upper curve log(N(M>M)), namely b=0.91, which leads to a global *q* value of 1.69 according to Equation (6). Notably, such *q* emphasizes the importance of Tsallis entropy (non-additive entropies in general) in this problem, since such *q* value is much larger than 1.0. The seismic phenomenon is clearly a non-equilibrium system, and the succession of events does not happen freely and spontaneously. On the contrary, the events are chained, beginning with a precursor that triggers the others nearby. If the events were independent, then the *q* value would tend to be 1.0 (Boltzmann–Gibbs entropy), which is not the case.

The sequence of the 55,853 magnitudes is illustrated in Figure 3. We can observe two transitions in the number of events close to days 1100 and 2190. The first transition coincides with the occurrence of the earthquake of magnitude 6.7, while the second transition is due to the two earthquakes with magnitudes 7.5 and 7.4 that happened on 6 February 2023. The details of these two transitions are presented in Figure 4.

### 5.1. Mutability on Magnitudes

**Figure 5:**
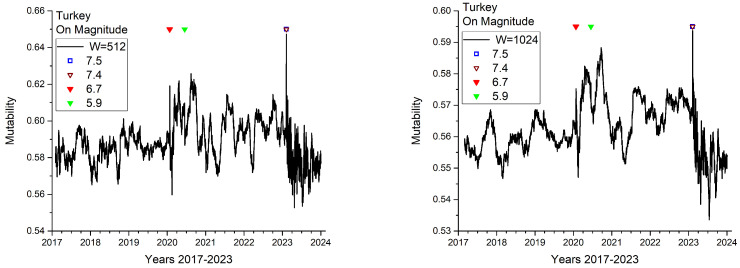
Mutability on the set of magnitudes for two time windows: (**Left**) Time window with 512 consecutive events. (**Right**) Time window with 1024 consecutive events.

Windows of 256, 512, 1024, and 2048 consecutive events were used to calculate dynamical mutability in magnitudes. The results for W=512 and W=1024 are shown in Figure 5. Special symbols in the upper part mark the positions of the earthquakes, as presented in Table 1. Some features are readily shown in this figure. Rather low mutability values with small oscillations are present until the end of 2020. The oscillations become larger and the values of mutability increase during the same year, following two large earthquakes. Then, the oscillations decrease throughout 2021, but the values remain somewhat larger than at the beginning of the 7-year period. A sharp upward “needle” marks the almost simultaneous 7.5 and 7.4 earthquakes. From then on, the mutability values decrease and strongly oscillate because of the aftershock regime that has not yet finished up to the point of collecting these data.

The curve for the window with 256 consecutive events (not shown) is like the one with 512 consecutive events, but noisier, making it difficult to identify the tendencies. The curve for the window with 2048 events is similar to the curve for W= 1024 consecutive events, but with less texture, which hides the differences as the main earthquakes approach. We believe that an analysis combining the windows with 512 and 1024 events can yield the appropriate information to appreciate the differences in time for the seismic dynamics in this area.

### 5.2. Mutability and Shannon Entropy

**Figure 6:**
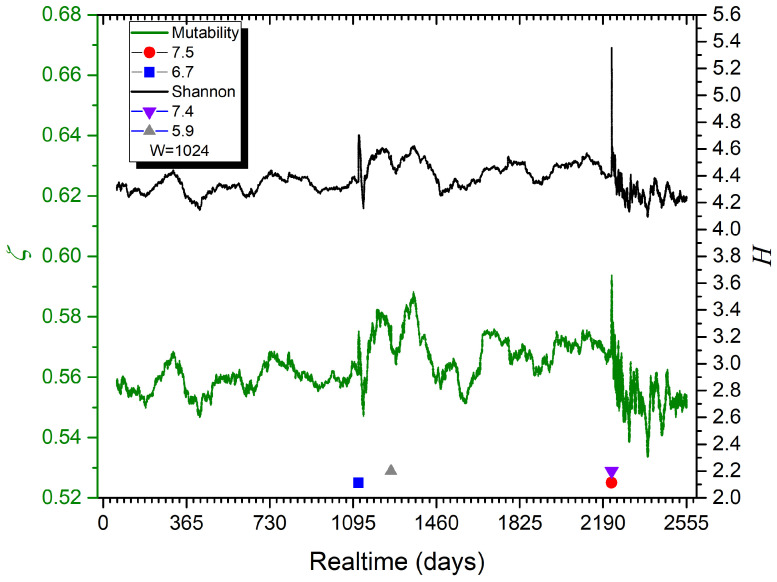
Mutability and Shannon entropy on magnitudes for successive overlapping time windows, each consisting of 1024 events. Symbols mark the earthquakes listed in Table 1.

Figure 6 presents the dynamical results based on magnitudes using mobile overlapping windows with 1024 consecutive events, showing both Shannon entropy (upper curve) and mutability (lower curve). The symbols representing the important earthquakes, as listed in Table 1, are included at two different levels to better appreciate the two earthquakes of 2023.

Initially, both curves in Figure 6 are very similar. Still, a careful comparison of these results shows that the mutability curve presents more texture and that the oscillations (signaling differences in the seismic activity) present a larger contrast. These differences can be associated with the dynamical properties of mutability that are not present in the Shannon entropy calculations based on histograms. One particular observation is that the larger oscillations in the mutability curve appear above the magnitude 6.4 earthquake, while the Shannon entropy remains flatter. Eventually, such dynamics reveal the precursor activity prior to the doublet of 2023. So, from now on, we will omit the Shannon entropy results, since the mutability curves present results with more contrast.

Both mutability and Shannon entropy present broad oscillations before the large earthquakes, followed by sharp decreasing oscillations during the aftershock period. When plotted with the same maximum vertical span, as in Figure 6, the mutability oscillations present larger amplitudes.

### 5.3. Tsallis Entropy

**Figure 7:**
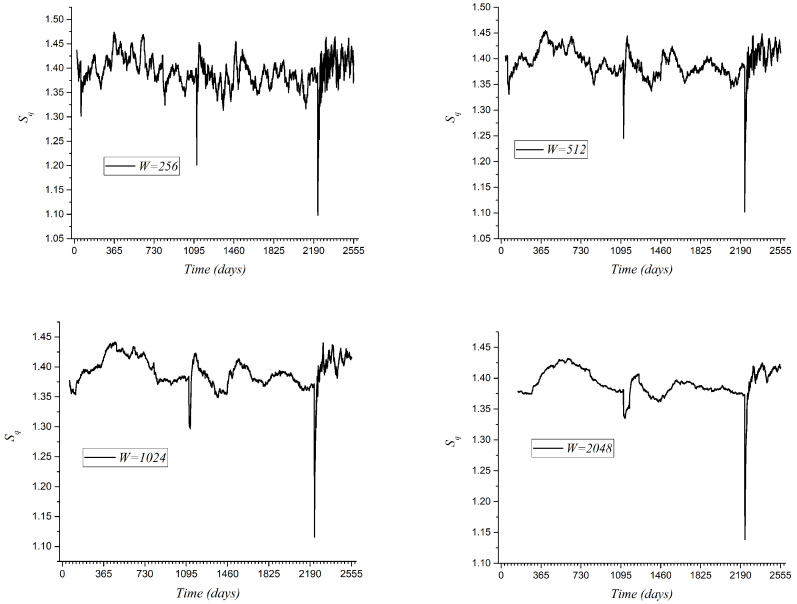
Dynamical Tsallis entropy for 4 different time windows W = 256, 512, 1024 and 2048 events, as indicated in the insets. Time is presented in blocks of 365 days to denote the years beginning with 2017.

Now, we present an analysis using Tsallis entropy on the magnitudes of the seismic events. This time, we present results using the four time windows with 256, 512, 1024, and 2048 consecutive events. These results are presented in Figure 7. As we found previously in the analysis of mutability and Shannon entropy, Tsallis entropy shows important variations due to the presence of the great earthquakes highlighted in Table 1. However, contrary to the mutability and Shannon entropy on magnitudes, these large earthquakes lead to downward needles in the Tsallis entropy curves. In this figure, we have omitted the special symbols for the earthquakes in Table 1, since their locations coincide exactly with the needles. Similarly to what was reported for the mutability on magnitude, for a window with 256 events, the curve is noisier than that of the curve for W=512. The curve for W=2048 shows less texture than the one for W=1024, and the first needle near day 1005 almost disappears.

Generally speaking, Tsallis entropy presents broad oscillations before the large earthquakes and sharp increasing oscillations during the aftershock period. Compared to the mutability curves, the Tsallis entropy curves recover faster after the main earthquakes.

### 5.4. Mutability and Tsallis

**Figure 8:**
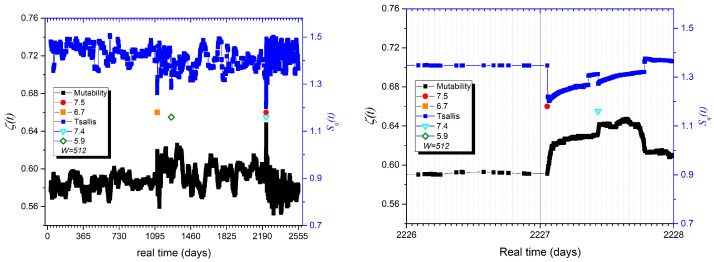
Combination of mutability (bottom, black) and Tsallis entropy (up, blue) for overlapping windows with 512 consecutive events. (**Left**) Full 7-year period; (**Right**) 2-day evolution: one day before and after the largest earthquake. Important earthquakes are indicated by special symbols defined in the insets.

In a previous work based on the seismicity of Iquique in Northern Chile [21], it was reported that a premonitory behavior was detected for both Tsallis entropy and mutability days and even hours before the main Mw 8.1 earthquake of 2014. To try to detect the possibility of prediction in the case of the Türkiye earthquakes, we prepared a similar treatment, which is presented in Figure 8. We include the Tsallis entropy (upper curve) and the mutability (lower curve) on the left-hand side. Both curves from the seven-year study are displayed in the left figure. The results show downward Tsallis needles coinciding with the upward mutability needles at the largest earthquakes. The right panel isolates just two days of the sequence, with the largest earthquake occurring in the early hours of the second day. As seen, both curves are flat on the previous day, meaning that the magnitudes of the earthquakes are very similar in the days prior to the earthquake. The only change happens at the moment of the large earthquake; no premonitory sign is found here. The previous “announcement” made by the 2014 earthquake in Iquique is likely because of a special behavior of the local dynamics, which does not necessarily accompany each earthquake.

The second earthquake barely shows in Figure 8, despite having almost the same magnitude as the first one. This is because the data from each earthquake are correlated with its own, but they are not inter-correlated. Therefore, the mixture of data within a short time interval significantly alters the cause-and-effect sequence.

## 6. Conclusions

The three entropic approaches used here render similar and consistent results. Shannon entropy and mutability are origin-related, since they are derived from the distribution of magnitudes within a period. However, mutability is dynamically constructed in such a way that it yields more texture than the Shannon entropy.

Tsallis entropy and mutability present opposite behaviors with respect to time evolution. Thus, important earthquakes produce downward needles in the Tsallis curves, while they produce upward needles in the mutability curves. Both approaches provide information about seismic energy release. Although the Tsallis entropy indicates an abrupt decrease with the occurrence of the main earthquake, which has been explained by Posadas and Sotolongo-Costas [5] through an asperity interaction model for earthquakes, the mutability indicates an increase in its value for the main shock. This increase in the value of mutability is related to the larger dispersion of magnitudes produced by the larger earthquake and its aftershock regime; the magnitudes of the aftershocks that occur after the main earthquake are less similar to each other, so the compression of data is lower and the mutability is larger. It would be beneficial if these two techniques were combined to better describe seismic activity.

To our knowledge, this is the first study on the significant seismic activity in Türkiye using three different entropy approaches. The largest recent activity, with the last aftershock regime still in the recovery process, indicates that this zone should be monitored in the future to detect any sign of important seismic activity.

The scarce seismic activity just prior to large earthquakes needs to be quantified. Unfortunately, the low number of earthquakes just before a large earthquake does not allow for a complete statistical treatment. Different tools should be used here, potentially a 3D network, to elucidate the characteristics of large earthquakes in each region.

Nevertheless, both mutability and Tsallis entropy clearly distinguish the foreshock and aftershock regimes. During the former, broad oscillations of the functions are the main characteristic, while during the latter, sharp increasing oscillations are appreciated for mutability, and sharp decreasing oscillations are appreciated for Tsallis entropy. 

## Figures and Tables

**Table 1:** Data for the four main earthquakes within the seismic zone marked in Figure 1.

Date	*M*	Latitude	Longitude
24 January 2020	6.7	38.3922 N	39.0847 E
14 June 2020	5.9	39.3353 N	40.7577 E
6 February 2023	7.5	37.1557 N	37.0850 E
6 February 2023	7.4	38.0818 N	37.1753 E

## Data Availability

Data are available on the websites of the Kandilli Observatory and Earthquake Research Institute (KOERI) and the Regional Earthquake and Tsunami Monitoring Center (RETMC) http://www.koeri.boun.edu.tr/new/en (accessed on 24 December 2024).
